# Serologic response following SARS-COV2 vaccination in patients with cancer: a systematic review and meta-analysis

**DOI:** 10.1186/s13045-022-01233-3

**Published:** 2022-02-05

**Authors:** Atsushi Sakuraba, Alexander Luna, Dejan Micic

**Affiliations:** Division of Gastroenterology, Hepatology and Nutrition, Department of Internal Medicine, The University of Chicago Medicine, 5841 S. Maryland Ave. MC 4076, Chicago, IL 60637 USA

**Keywords:** COVID-19, Vaccine, Outcomes, Meta-analysis, Cancer, Immunocompromised

## Abstract

**Purpose:**

Patients with cancer have an increased risk of coronavirus disease 2019 (COVID-19) and an attenuated responses to various vaccines. This meta-analysis aims to assess the serologic response to COVID-19 vaccination in patients with cancer.

**Methods:**

Electronic databases were systematically searched on August 1, 2021 for studies that reported the serologic response to COVID-19 vaccine in cancer patients. Random effects models were used to achieve pooled serologic response rates and odds ratios (ORs).

**Results:**

We analyzed 16 observational studies with a total of 1453 patients with cancer. A majority of studies used mRNA vaccines (BNT162b2 or mRNA-1273). The proportion of patients achieving a serologic response after a single and two doses of COVID-19 vaccine were 54.2% (95% confidence interval [CI] 41.0–66.9) and 87.7% (95% CI 82.5–91.5), respectively. Patients with hematologic cancers had a lower response rate after the second dose of vaccine compared to those with solid organ cancers (63.7% vs. 94.9%), which was attributable to the low response rates associated with certain conditions (chronic lymphocytic leukemia, lymphoma) and therapies (anti-CD20, kinase inhibitors). A lower proportion of patients with cancer achieved a serologic response compared to control patients after one and two doses of vaccine (OR0.073 [95% CI 0.026–0.20] and 0.10 [95% CI 0.039–0.26], respectively).

**Conclusions:**

Patients with cancer, especially those with hematologic B-cell malignancies, have a lower serologic response to COVID-19 vaccines. The results suggest that cancer patients should continue to follow safety measures including mask-wearing after vaccination and suggest the need for additional strategies for prophylaxis.

**Supplementary Information:**

The online version contains supplementary material available at 10.1186/s13045-022-01233-3.

## Introduction

The coronavirus disease 2019 (COVID-19) pandemic caused by severe acute respiratory syndrome coronavirus 2 (SARS-CoV-2) persists and the world is seeing another year of a global health emergency [1]. Extensive research over the past year has demonstrated that elderly patients as well as those with pre-existing conditions such as obesity, diabetes, and cancer are more susceptible to getting infected with SARS-CoV-2 and developing life threatening pneumonia [2–4]. Approximately 9.5% of the adult population in the USA have or have had a diagnosis of cancer of any type and 1 in 3 adults will be diagnosed with cancer at some point during their lifetimes [5, 6]. Protecting the health and safety of patients with cancer is vital and considered a major priority during the COVID-19 pandemic [7].

Mehta et al. reported that increased age, comorbidities, and poor performance status were associated with poor outcome to COVID-19 in patients with cancer [4]. Case fatality rates (CFR) were greater in patients with hematologic cancers compared to those with solid cancers (37% vs. 25%) [4]. These data suggest the need for prophylactic strategies in cancer patients, who are often immunosuppressed due to their underlying disease state and use of chemotherapy, radiation therapy, bone marrow or stem cell transplant, and/or immunotherapy. With the lack of effective treatments for COVID-19, prevention strategies including vaccination are of paramount importance in reducing the risk and mortality [8]. Vaccines against COVID-19 were rapidly developed and emerging data shows that mRNA-based COVID-19 vaccines are effective and safe in the general population. However, the efficacy of COVID-19 vaccines in patients with cancer is unknown as patients with active cancer or those treated with immunosuppressing therapies were excluded from regulatory vaccine trials [9]. Guidelines currently recommend that patients with cancer should be vaccinated against SARS-CoV-2 as long as any components of the vaccine are not contraindicated [10]. Data from other vaccine-preventable illnesses suggest an attenuated humoral response to vaccines in patients with solid cancer undergoing cytotoxic chemotherapy [11, 12]. Recent studies have reported that patients with hematologic malignancies, especially those receiving anti-CD20 therapies or those with a history of stem cell transplantation had attenuated immunogenicity to BNT162b2 or mRNA-1273 SARS-CoV-2 vaccines compared to those with solid tumors [13, 14]. Additional studies investigating the effectiveness of COVID-19 vaccines in cancer patients are limited and mostly are of small sample sizes.

Better understanding of the overall effectiveness of COVID-19 vaccines in patients with cancer would improve clinical care and protect these vulnerable patient population. In this systematic review and meta-analysis, our aim was to integrate findings across studies to determine the serologic response rate to COVID-19 vaccination in patients with cancer.

## Methods

### Search strategy and study selection

This meta-analysis was conducted according to a *priori* defined protocol that is in accordance with the PRISMA guideline [15]. The protocol of this meta-analysis has been submitted to the International Prospective Register of Systematic Reviews (PROSPERO) [16]. We searched PubMed/MEDLINE, EMBASE, and medRxiv (https://www.medrxiv.org/) from inception to August 1, 2021 to identify studies assessing the response to COVID-19 vaccination in patients with cancer.

**Fig. 1:**
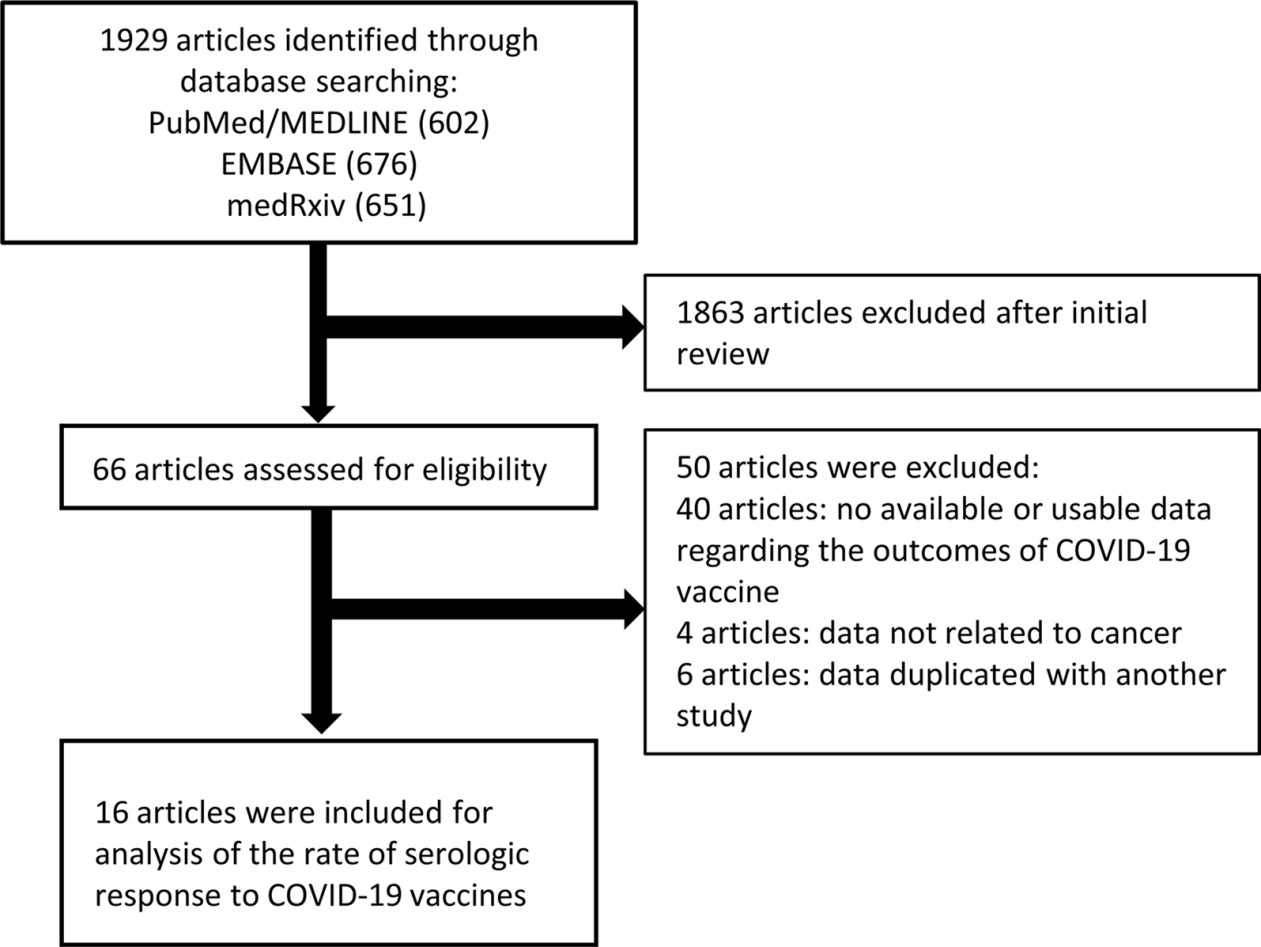
Flowchart of the assessment of the studies identified in the meta-analysis

We included observational studies reporting the outcomes of COVID-19 vaccination in patients with cancer. There were no restrictions regarding age, sex or duration of the study. Studies reporting outcomes in patients with active or history of cancer were eligible. We imposed no geographic or language restrictions. Two authors (AL, AS) independently screened each of the potential studies to determine whether they were eligible for inclusion. Areas of disagreement or uncertainty were resolved by consensus among the authors. Studies were identified with the following terms:”COVID-19″, “SARS-CoV-2″, “vaccine”, “cancer”, “malignancy”, “leukemia”, “lymphoma”, “immunosuppressed”, and “hematologic diseases”. A search was also performed of bibliographies of identified articles for additional references. The search was restricted to human studies. Manuscripts published in languages other than English were translated if necessary. Single case reports were excluded. Studies that reported only adverse outcomes to COVID-19 vaccination were excluded. The corresponding authors of studies were contacted to obtain additional information when needed. The search strategy is described in Fig. 1 and the Pubmed/MEDLINE, EMBASE, and medRxiv search strategies are shown in Additional file 1: Table S1.

### Data extraction and quality assessment

All data were independently abstracted in duplicate by two authors (AL, AS) by using a data extraction form. Data on the study characteristics including author name, year of publication, study design, duration, study location, sample size, diagnosis of cancer, concomitant medication use, age and gender of patients, type and frequency of vaccination, and type and outcome of serologic testing were collected. We divided diseases into the following two categories: (1) solid tumors, and (2) hematologic malignancies. Proportion of patients undergoing active cancer therapies and using glucocorticoids (GCs) were extracted when data was available.

We assessed the risk of bias of included studies using the Joanna Briggs Institute Critical Appraisal Checklist [17]. We rated the quality of evidence according to the Grades of Recommendation, Assessment, Development and Evaluation (GRADE) approach to assess the certainty of evidence obtained from the present meta-analysis [18].

### Outcome assessment

The primary outcome was the rate of serologic response to COVID-19 vaccination in cancer patients. Response was separately assessed after one or two vaccinations. The secondary outcome of interest was the rate of serologic response in cancer patients compared to control patients. We extracted the number of patients who achieved an above cut-off antibody level among the total number of patients tested in each study. Applying a common cut-off value between studies was not possible because each study used a different serologic testing method. When tests were performed at multiple time points in a study, we chose the date closest to 4 weeks after the vaccination.

Subgroup analyses or meta-regression according to cancer type (hematological vs solid cancer), proportion of patients on anti-cancer medication/treatment, and age of subjects were undertaken when data were available. Anti-cancer medications/treatments included chemotherapy, monoclonal antibodies, immune check-point inhibitors, radiotherapy, hormonal therapy, etc. and were assessed separately.

### Statistical analysis

We undertook a meta-analysis of the rate of serologic response to COVID-19 vaccination among individuals with cancers from observational studies by using a random effects model. The presence of heterogeneity across studies was assessed by using the *I*^2^ statistic. An *I*^2^ value of < 25% indicates low heterogeneity, 25–75% as moderate heterogeneity and > 75% as considerable heterogeneity [19]. Heterogeneity was evaluated by using Cochran’s Q-statistics with a significance level of *P* < 0.10 [20]. Begg’s and Egger’s tests were performed to assess publication bias and funnel plots were constructed to visualize asymmetry when ≥ 3 studies were available [21, 22]. A random effects meta-regression model was used to assess the contributions of each potential risk factors and medication class to the outcome of vaccine response. When the number of available studies for each analysis was less than 10, funnel plot construction and meta-regression analysis were undertaken for reference purpose due to its low reliability.

We included preprints because they form a substantial part of the available COVID-19 evidence, but due to their lack of peer-review, we conducted a sensitivity analysis by excluding preprints [23]. Four studies included non-mRNA type vaccines, so we conducted a sensitivity analysis by excluding them. We also performed one study removed analyses to assess whether the results are strongly influenced by any single study.

Statistical analyses were performed using the Comprehensive Meta Analysis Software (version 3; Biostat, Englewood, NJ, USA). All statistical tests except for the Q-statistics used a two-sided *P* value of 0.05 for significance.

### Data sharing and access

Data will be made available upon request to the corresponding author. All authors had access to the study data and reviewed and approved the final manuscript.

## Results

### Study characteristics

We identified 1929 citations through the literature search, excluded 1863 after initial title and abstract screening, and assessed 66 studies for eligibility. Sixteen articles including 1453 patients met eligibility criteria (Fig. 1). As shown in Table 1, seven were full-text articles [13, 14, 24–28], seven were correspondence/letters [24, 29–34], and two were articles in a preprint [35, 36]. Four studies included only patients with solid organ cancers, nine included only hematologic cancers, and three studies included patients with both solid and hematologic cancers. The three studies that included both types of cancers reported seroconversion rates separately among the types of cancers, so we reported them separately. Eight studies used only BNT162b2 (Pfizer-BioNTech), four studies used BNT162b2 and mRNA-1273 (Moderna), two studies used ChAdOx1 nCoV-19 (Oxford-AstraZeneca) and BNT162b2, one study used ChAdOx1 nCoV-19, mRNA-1273, and BNT162b2, and one study used AD26.COV2.S (Janssen/Johnson & Johnson), mRNA-1273, and BNT162b2. The majority of the studies that used non-mRNA type vaccines did not report outcomes separately, so we reported them together with the mRNA vaccination strategies.

**Table 1:** Characteristics and outcomes of the included studies

Author	Country	Year	Patient numbers and description	Control numbers and description	Age of patients (years)	Sex of patients (%females)	Cases, % of patients on anti-cancer therapy	Cases, % of patients on steroids	Type of vaccine	Number of patients receiving 1 dose	Number of patients receiving 2 doses
Monin	United Kingdom	2021 Full-text article	95 solid cancers (Women’s cancers 35%, Urological cancers 16%, Skin cancers 13%, Thoracic cancers 22%, Gastrointestinal cancers 13%, etc.), 56 hematological cancers (Mature B-cell neoplasms 68%, Mature T-cell neoplasms 9%, Myeloid and acute leukemia neoplasms 18%, etc.)	54 (Healthy controls)	Cases 73 (19–94), Controls 40.5 (22–78)	Cases 48%, Controls 48%	Solid cancers: Chemotherapy 31.5%, CPI 14.1%, Chemotherapy + CPI 6.5%, Targeted therapies 14.1%, Endocrine therapies 12.0%, Radiotherapy 5.4%, Surgery 8.7% Hematological cancers: Chemotherapy 10.9%. Targeted therapies 16.4%, Single-agent monoclonal antibodies 3.6% Chemo/targeted therapies + immunotherapy 27.3%, Lenalidomide 7.3%, CPI 1.8%, Radiotherapy 9.1%, Surgery 1.8%	NA	BNT162b2 (Pfizer-BioNTech) 100%	Cases 100 (Solid cancers 56, hematological cancers 44) Controls 34	Cases 24 (Solid cancers 19, hematological cancers 5) Controls 12
Harrington 1	United Kingdom	2021 Full-text article	16 hematological cancers (Chronic phase CML 100%)	None	Median: 45 (Range 23–74)	25%	Targeted therapy 93.75%, targeted therapy + HSCT 6.25%	NA	BNT162b2 (Pfizer-BioNTech) 100%	16	NA
Bird	United Kingdom	2021 Comment	93 hematological cancers (Multiple myeloma 100%) (Disease status (per IMWG criteria): Complete response or very good partial response 51.6%, Partial response 17.2%, Stable disease or progressive disease 29.0%, Unable to assess 2.15%)	None but reports 177 hospital staff getting the same test	67 (IQR 59–73)	40.9%	Immunomodulatory drugs 66.7%, PI 27.3%, Anti-CD38 antibody 31.8%, Other therapy 15.2%	63.6%	Cases: BNT162b2 (Pfizer-BioNTech) 51.6% AZD1222 (Oxford-AstraZeneca) 48.4% Controls NA	93	NA
Herishanu	Israel	2021 Full-text article	167 hematological cancers (CLL or SLL 100%) (Treatment-naïve: 34.7%, on-therapy: 44.9%, off-therapy in remission: 14.4%, off-therapy in relapse: 6.0%)	52 (Healthy age- and sex-matched controls)	Case median: 71.0 (IQR 63.0–76.0), Control median: 68.0 (IQR 64.0–74.0)	32.9%	BTK inhibitors 66.7%, Venetoclax ± anti-CD20 antibodies 29.3%, Other 4.0%	NA	BNT162b2 (Pfizer-BioNTech) 100%	NA	219
Agha	United States	2021 Preprint	67 hematological cancers (B-cell chronic lymphocytic leukemia 19.4%, other lymphomas 31.3%, multiple myeloma 43.3%, other myeloid malignancies 5.97%)	None	Median: 71 (IQR 65–77)	47.8%	Thalidomide derivatives 20.4%, Proteasome inhibitors 18.4%, Monoclonal antibodies 22.5%, Tyrosine kinase inhibitors 14.3%, Other therapies 4.1%	20.4%	mRNA-1273 (Moderna) 41.8% BNT162b2 (Pfizer-BioNTech 50.8%, unavailable 7.4%	NA	67
Roeker	United States	2021 Letter to the editor	44 hematological cancers (CLL 100%)	None	Median: 71 (Range 37–89)	47.8%	BTK inhibitor 32%, Venetoclax 16%, anti-CD20 antibodies within 1 year 32%	NA	BNT162b2 (Pfizer-BioNTech) 57% mRNA-1273 (Moderna) 43%	NA	44
Thakkar	United States	2021 Full-text article	134 solid cancers (Breast 38.1%, Gastrointestinal 20.1%, Genitourinary 13.4%, Gynecological 7.5%, Thoracic/head & neck 18.7%, Skin/musculoskeletal 1.5%, Carcinoma of unknown primary 0.7%), 66 hematological cancers (Lymphoid 39.4%, Myeloid 27.3%, Plasma cell 33.3%)	26 (Healthy controls)	Case median: 67 (Range 27–90), Control median: 64 (Range 37–82)	Cases: 58%, Control: 62%	Chemotherapy 56%, Targeted therapy 19.5%, Immunotherapy 54%, Hormonal therapy 23.5%, Experimental therapy 3.5%, Protease inhibitor 9.5%, Antibody–drug conjugate 3.5%	NA	Cases: BNT162b2 (Pfizer/BioNTech) 58% mRNA-1273 (Moderna) 31%, AD26.COV2.S (Janssen/Johnson & Johnson) 10% mRNA (type unknown) 2% Controls: NA	Cases: 20, Controls: NA All data reported after final dose in vaccination series	Cases: 180 (Solid cancers 134, hematological cancers 66), Controls: 26
Palich	France	2021 Letter to the editor	223 solid cancers (Breast 40%, Digestive 16%, Lung 14%, Gynecological 11%, Prostate 4.0%, Bladder 3.6%, Pancreas 3.6%, Kidney 2.7%, Upper aero-digestive tract 2.7%, Others 3.1%)	49 (Healthy volunteers)	Case median: 67 (IQR 60–75), Control median: 53 (IQR 47–60)	Cases: 64%, Controls: 65%	Chemotherapy 58%, Targeted therapy 35%, Immunotherapy 13%, Hormone therapy 12%, Radiotherapy 2.2%	NA	BNT162b2 (Pfizer/BioNTech) 100%	NA	Cases: 223, Controls: 49
Chowdhury	United Kingdom	2021 Letter to the editor	59 hematological cancers (Chronic myeloid leukemia 20.3, Essential thrombocythemia 27.1%, Myelofibrosis 11.9%, Polycythemia vera 18.6, Myelodysplastic syndrome 22.0%)	232 (Healthcare workers > 60 years old)	Case median: 62 (IQR 52–73), Control median: 62 (Range 60–76)	Cases: 54.2% Control: NA	Chemotherapy 23.7%, Immunotherapy 13.6%, Targeted therapy 28.8%, Chemotherapy + targeted therapy 1.7%, BET inhibitor 1.7%, Darbopoietin 5.1%, Anagrelide 1.7%	NA	Cases: BNT162b2 (Pfizer-BioNTech) 37.3% AZD1222 (Oxford-AstraZeneca) 62.7% Controls: BNT162b2 (Pfizer-BioNTech) 72.8%, AZD1222 (Oxford-AstraZeneca) 27.2%	Cases: 59, Controls: 232	NA
Diefenbach	United States	2021 Preprint	53 hematological cancers (CLL/SLL 32%, Hodgkin lymphoma 8%, DLBCL 19%, Follicular lymphoma 13%, Mantle cell lymphoma 6%, Marginal zone lymphoma 13%, Low-grade B cell lymphoma 2%, Burkitt lymphoma 2%, Waldenstrom's macroglobulinemia 6%)	5 (Healthy vaccinated controls: 3, Convalescent vaccinated controls: 2)	Case median: 63 (Range 25–98), Control: NA	Cases: 47%, Control: NA	Anti-CD20-based therapy 45%, BTK inhibitors 19%, Chemotherapy 8%	NA	Cases: BNT162b2 (Pfizer-BioNTech) 77% mRNA-1273 (Moderna) 23% Controls: NA	Cases: 52 Data from first and second vaccinations were not reported separately	Cases: 19, Controls: 5
Terpos	Greece	2021 Letter to the editor	59 solid cancers (Lung 27.1%, Bladder 25.4%, Kidney 18.6%, Uterine 8.5%, Pancreatic 5.1%, Other 13.6%)	283 (Healthy controls)	Case median: 66 (IQR 61–76), Control median: 64 (IQR 59–82)	Cases: 34.8%, Control: "We used case–control matching to match the two groups for age, gender, and type of vaccine with the calipmatch command in Stata."	Immune checkpoint inhibitors 96.6%, I/O combination 3.4%	NA	Cases: BNT162b2 (Pfizer-BioNTech) 69.5% AZD1222 (Oxford-AstraZeneca) 25.4% mRNA-1273 (Moderna) 5.1% Controls: mRNA vaccine (BNT162b2 or mRNA-1273) 82%, AZD1222 (Oxford-AstraZeneca) 18%	Cases: 59, Controls: 283	NA
Harrington 2	United Kingdom	2021 Letter to the editor	21 hematological cancers (Essential thrombocythaemia 38.1%, Myelofibrosis 42.9%, Polycythaemia vera 19.0%)	None	Mean: 55 (Range 36–72)	66.7%	Chemotherapy 19.0%, Immunotherapy 19.0%, Targeted therapy 23.8%, Targeted therapy + IMG-7289 4.8%	NA	BNT162b2 (Pfizer-BioNTech) (100%)	21	NA
Pimpinelli	Italy	2021 Full-text article	92 hematological cancers (Multiple myeloma 45.7%, MPM 54.3%)	36 (Healthy controls)	Multiple myeloma median: 73 (Range 47–78), MPM median: 70 (Range 28–80), Control median: 81 (Range 79–87)	Multiple myeloma: 45.2%, MPM: 48%, Control: 50%	Chemotherapy 21.7%, PI-based combination therapies 9.8%, Immunomodulatory imide drugs 20.7%, Targeted therapy 28.3%	45.7%	BNT162b2 (Pfizer-BioNTech) (100%)	NA	Cases: 92, Controls: 36
Addeo	United States, Switzerland	2021 Full-text article	106 solid cancers (Breast 25.5%, Urological 18.9%, Gynecological 2.8%, Skin 6.6%, Thoracic malignancy 17.0%, Gastrointestinal 15.1%, Head and neck 2.8%, Brain 7.5%, Connective tissue 3.8%) 25 hematological cancers (Diffuse large B cell lymphoma 24%, Follicular lymphoma 8%, MALT lymphoma 8%, T-cell Lymphoma/Mycosis Fungoides 2, 8%, Hodgkin’s lymphoma 16%, Polycythemia Vera 4%, Myeloma 20%)	None	Median: 63 (IQR 55 – 69)	45%	Cytotoxic chemotherapy 23%, Immunotherapy 11%, Endocrine therapy 15%, Targeted therapies 25%, Unknown (double-blind clinical trial) 1%	NA	BNT162b2 (Pfizer-BioNTech) 29% mRNA-1273 (Moderna) 71%	Cases: 121	Cases: 123
Barrière	France	2021 Letter to the editor	122 solid cancers	29 (Healthy volunteers)	Cancer median: 69.5 (Range 44–90), Control median: 53 (Range 21–81)	Cancer: 47.5%, Control: NA	Chemotherapy (CT) ± targeted therapy 86.0%	NA	BNT162b2 (Pfizer-BioNTech) (100%)	Cases: 122, Controls: 13	Cases: 42, Controls: 24
Massarweh	Israel	2021 Full-text article	102 solid cancers (Gastrointestinal 28%, Lung 25%, Breast 18% cancer, Brain 9%, Genitourinary 8% Other 12%)	78 (Family members/caregivers of case patients)	Case median: 66 (Range 56–72) Control median: 62 (Range 49–70)	Cancer: 43%, Control: 68%	Chemotherapy 29%, Immunotherapy 22%, Chemotherapy + biological therapy 20%, Chemotherapy + immunotherapy 14%, Biological therapy 11%, Immunotherapy + biological therapy 5%	NA	BNT162b2 (Pfizer-BioNTech) (100%)	NA	Cases: 102, Controls; 78

Author	Test used to check antibody response	Timing of test	After one dose				After two doses			
			One dose				Two doses			
			Cases responders	Controls responders	Cases Ab titers	Controls Ab titers	Cases responders	Controls responders	Cases Ab titers	Controls Ab titers
Monin	IgG antibodies to SARS-CoV-2 spike (S) protein, ELISA	3 and 5 weeks after the first vaccination (second vaccination administered at 3 weeks)	29/100 (Solid cancers 21/56, hematological cancers 8/44) Women’s cancers 9/19, Urological cancers 4/6, Skin cancers 2/9, Thoracic cancers 3/14, Gastrointestinal cancers 3/6) Mature B-cell neoplasms 6/31 (CLL 1/6, Lymphoma 2/15, MM 3/9), Mature T-cell neoplasms (Anaplastic large cell lymphoma) 1/4, Myeloid and acute leukemia neoplasms 1/8 (AML 0/1, CML 0/2, MDS/MPM 1/2)	32/34	–	–	21/24 (Solid cancers 18/19, hematological cancers 3/5) Women’s cancers 6/6, Urological cancers 1/1, Skin cancers 4/5, Thoracic cancers 3/3, Gastrointestinal cancers 4/4 Mature B-cell neoplasms 2/4 (CLL 1/2, Lymphoma 0/1, MM 1/1), Myeloid and acute leukemia neoplasms 1/8 (MDS/MPM 1/1)	12/12	–	–
Harrington 1	Anti-S IgG ELISA	Median 21 days (IQR 21–27.25) after first vaccination	14/16 (CML)	–	Median EC50: 100.5 (IQR 25–408.3)	–	–	–	–	–
Bird	Ortho Clinical Diagnostic Anti-SARS-CoV-2 IgG and Anti-SARS-CoV-2 Total chemiluminescent immunoassays	Median 33 days (IQR 28–38) after first vaccination	Total: 52/93 (MM) BNT162b2: 26/48, AZD1222: 26/45	175/177 HCWs	NA	NA	–	–	–	–
Herishanu	Roche Diagnostics Elecsys® Anti–SARS-CoV-2S assay	Median 15 days (IQR 14–17) after second vaccination	–	–	–	–	66/167 (CLL) Active tx 12/75 (BTKinh 8/50, venetoclax + / − anti-CD20 3/22), No tx 54/92	52/52	Median: 0.824 U/mL (IQR 0.4–167.3, including 155 U/mL (IQR 7.6–490.3))	Median: 1084 U/mL (IQR 128.9–1879)
Agha	Anti-RBD IgG antibody assays Reactive results confirmed by the Siemens SARS-CoV-2 Total Ig Assay, which detects both IgM and IgG antibodies against RBD 35 of the S1 subunit of the Spike protein	Median 23 days (IQR 16–31) after second vaccination	–	–	–	–	36/67 (CLL 3/13, Lymphoma 11/21, MM 19/29, AML/CML 3/4) Active tx 15/30, No tx 21/37 BNT162b2: 15/34, mRNA-1273: 16/28	–	Responders: Median antibody level: 14.42 (IQR 3.31–25.58), Non-responders: Median antibody level: 0.03	–
Roeker	DiaSorin Liaison® SARS-CoV-2 S1/S2 IgG assay	Median 21 days (range 14–48) after second vaccination	–	–	–	–	23/44 (CLL) Active tx 6/26 (BTKinh 3/14, anti-CD20 2/14, venetoclax/anti-CD20 0/7), No tx 17/18	–	NA	–
Thakkar	Abbott AdviseDx SARS-CoV-2 IgG II chemiluminescent immunoassay	Solid cancers: median 31.5 days after final vaccination, hematological cancers: median 28.5 days after final vaccination, Controls: median 30 days (IQR 19–53 days) after final vaccination	–	–	–	–	Total: 187/200 (Solid cancers 131/134, hematological cancers 56/66) Active tx 120/125 (Anti-CD20 16/23, SCT 19/26, CAR-T 0/3, Hormonal tx 47/47, ICI 30/31), No tx 55/59 BNT162b2: 109/115, mRNA-1273: 58/62, Ad26.COV2.S: 17/20	26/26	BNT162b2: Median 5173 AU/mL (SD 16,699), mRNA-1273: Median 11,963 AU/mL (SD 18,742), Ad26.COV2.S: Median 1121 AU/mL (SD 17,571)	NA
Palich	Abbott Elity SARS-CoV-2 IgG chemiluminescent microparticle immunoassay (CMIA), Roche Elecsys SARS-CoV-2 total Ig electrochemiluminescent immunoassay (ECLIA), or other immunoassays	Cases: Median 25 days (IQR 21–29) after second vaccination, Controls: Median 8 days (IQR 7–14) after second vaccination	–	–	–	–	210/223	49/49	Roche median: 252 UA/mL Abbott median: 4443 U/ML	Roche median: 2517 UA/mL Abbott median: 13,285 U/mL
Chowdhury	Abbott SARS-CoV-2 IgG II Quant Assay for anti-S titers	Cases: median 34 days (IQR 28–56) after first vaccination, Controls: > 14 days after first vaccination	Total: 34/59 CML 9/12, ET 11/17, PV 5/11, MF 4/7, MDS 6/13 Active tx 27/47 (IFN 7/8, Ruxolitinib 0/4, Hydroxycarbamide 4/11), No tx 7/12 BNT162b2: 12/22 ADZ122: 22/37	224/232	Median: 75 AU/mL (IQR 19–328)	Median: 630 AU/mL (IQR 284–1328)	–	–	–	–
Diefenbach	Multiplex bead–binding assay	2 weeks (± 1 week) after the first vaccination and 6 weeks (± 2 weeks) after the second or single dose vaccination	–	–	–	–	25/53 Active tx 14/38 (BTKinh 4/10, Anti-CD20 6/24, Chemotx 4/4), No tx 11/15	5/5	NA	Healthy vaccinated controls mean RBD IgG: 1.03 × 10^5^ Convalescent vaccinated controls mean RBD IgG: 5.29 × 10^4^
Terpos	GenScript cPass SARS-CoV-2 NAbs Detection Kit	22 days after first vaccination	15/59	186/283	Median NAb inhibition titer: 22% (IQR 13.4–30.2%)	Median NAb inhibition titer: 38% (IQR 23–54%)	–	–	–	–
Harrington 2	Anti-S IgG ELISA	Median 21 days (IQR 21–21) after first vaccination	Total: 16/21 ET 8/8, MF 6/8, PV ¼ Active tx 12/14 (IFN 4/4, Ruxolitinib 4/6, Hydroxycarbamide 4/4), No tx 4/7	–	Median anti-S IgG EC50: 239 (IQR 25–4544)	–	–	–	–	–
Pimpinelli	DiaSorin LIAISON SARS-CoV-2 S1/S2 IgG quantitative chemiluminescent immunoassay (CLIA)	21 and 35 days after first vaccination	35/92 (MM 9/42, MPM 26/50)	19/36	Multiple myeloma: Geometric mean concentration 7.5 AU/mL (95% CI 5.6–10.4) MPM: Geometric mean concentration 16.2 AU/mL (95% CI 11.7–22.3)	Geometric mean concentration: 17.1 AU/mL (95% CI 12.0–24.1)	77/92 (MM 33/42, MPM 44/50) Daratumumab-based 7/14, PI/IMid based 26/28	36/36	Multiple myeloma: Geometric mean concentration 106.7 AU/mL (95% CI 62.3–179.7) MPM: Geometric mean concentration 172.9 AU/mL (95% CI 106.5–257.0)	Geometric mean concentration: 353.3 AU/mL (95% CI 255.6–470.0)
Addeo	Roche Diagnostics Elecsys Anti-SARS-CoV-2S immunoassay for antibodies (including IgG) to the SARS-CoV-2 spike (S) protein receptor binding domain (RBD)	Median 24 days (Range 22 – 24) after second vaccination	98/121 (Solid cancers 80/96, hematological cancers 18/25) Active tx 60/77 (Kinase inh 13/15, Anti-CD20 0/4, Chemotx 20/29, ICI 11/13), No tx 38/44 BNT162b2: 24/29, mRNA-1273: 74/92	– –	Median 32 U/mL (IQR 2–105)	–	116/123 (Solid cancers 99/101, hematological cancers 17/22) Active tx 72/78 (Kinase inh 12/13, Anti-CD20 0/4, Chemotx 28/30, ICI 13/14), No tx 44/45 BNT162b2: 28/30, mRNA-1273: 88/93	–	Median 2500 U/mL (IQR 438–2500)	–
Barrière	Roche Diagnostics Elecsys Anti-SARS-CoV-2 immunoassay	3–4 weeks and 6–8 weeks after baseline	58/122 Chemotx 45/105, targeted tx 13/17	13/13	Median 0.52 UI/mL (range 0–1962)	Median: 21.6 UI/mL (range 3.26–723.2)	40/42	24/24	Median: 245.2 UI/mL (range 0–5467)	Median: 2517 UI/mL (range 157.6–6318.0)
Massarweh	Abbott SARS-CoV-2 IgG II Quant chemiluminescent microparticle immunoassay	Cases: median 38 days (IQR 32–43) after second vaccination, Controls: median 40 days (32–4) after second vaccination	–	–	–	–	92/102 Women’s cancers 6/9, Urological cancers 7/8, Brain 9/9, Thoracic cancers 24/26, Gastrointestinal cancers 25/29, Other 12/12	78/78	Median: 1931 AU/mL (IQR 509–4386)	Median: 7160 AU/mL (IQR 3129–11,241)

*ICI* immune checkpoint inhibitor, *CML* chronic myeloid leukemia, *HSCT* hematopoietic stem cell transplantation, *CLL* chronic lymphocytic leukemia, *SLL* small lymphocytic lymphoma, *MPM* myeloproliferative malignancies, *PI* proteasome inhibitor, *IFN* interferon, *IMiD* immunomodulatory drug, *HCW* health care worker, *NA* not available

For the analyses on the rate of serologic response to COVID-19 vaccination, nine and eleven studies were available for assessment after one and two doses, respectively. Six and eight studies compared outcomes after one or two doses of COVID-19 vaccine to a control population without cancers. A majority of the studies assessed serologic response 3–5 weeks post-vaccination. All studies included for the assessment of response after two doses did not delay the timing of the second dose. The summary of characteristics and outcomes of the included studies are summarized in Table 1. The risk of bias of included studies assessed using the Joanna Briggs Institute Critical Appraisal Checklist is shown in Additional file 1: Table S2. A majority of the studies were of medium to high quality.

### Rate of serologic response after a single dose of COVID-19 vaccine

**Fig. 2:**
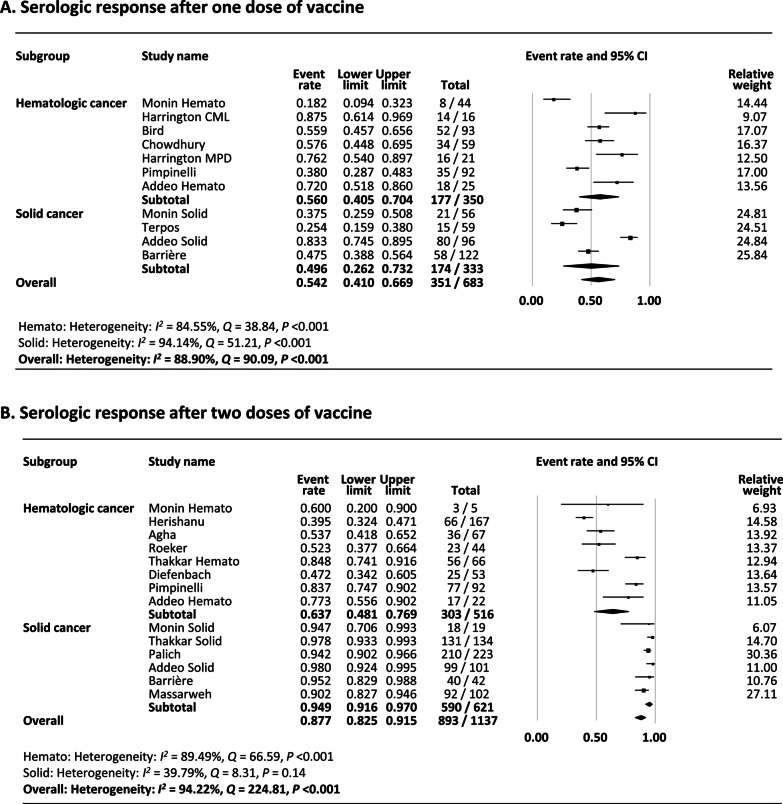
**A** Meta-analysis of serological response after one dose of vaccine. **B** Meta-analysis of Serological response after two doses of vaccine

There were nine studies (11 reports) that assessed the serologic response after the first dose of COVID-19 vaccine in patients with cancer [14, 24, 25, 27, 29, 32–34] The studies by Monin et al. and Addedo et al. reported outcomes separately in hematologic and solid cancers [14, 25]. As shown in Fig. 2A, the pooled proportion of patients achieving a serologic response was 54.2% (95% confidence interval [CI] 41.0–66.9). Subgroup analysis demonstrated that the rate was numerically lower in solid cancers (49.6%) compared to hematologic cancers (56.0%).

Heterogeneity was present (*I*^2^ = 88.9%) likely attributed to the difference in the reported rates among studies. Multivariate meta-regression was undertaken to further explore the cause of heterogeneity and demonstrated that the proportion of patients on anti-cancer therapy (coefficient − 0.029, 95% CI − 0.055 − (− 0.0016), *P* = 0.038) and the age of the subjects (coefficient − 0.10, 95% CI − 0.16 − (− 0.050), *P* < 0.001) were significant sources of heterogeneity, with studies with more patients on anti-cancer therapy and with older subjects presenting a lower serologic response rates to a single dose of COVID-19 vaccine (Additional file 1: Table S3).

Funnel plot of the studies included in the meta-analysis demonstrated no asymmetry and no publication bias was found (Begg’s *P* = 0.44, Egger’s *P* = 0.48) (Additional file 1: Fig. S1A).

Sensitivity analyses were carried out to assess whether individual studies influenced the results (Additional file 1: Fig. S1B). When individual studies were removed one at a time from the analyses, the corresponding pooled rates were not markedly altered by any single study confirming the stability of the results. Three studies included non-mRNA type vaccines [29, 32, 34] and a sensitivity analysis excluding them showed similar results (Additional file 1: Fig. S1C). No preprint studies were included for this analysis, so sensitivity analysis excluding preprints was not performed.

Subgroup analysis according to type of hematologic cancer demonstrated that rates were lower in chronic lymphocytic leukemia (CLL) (16.7%), and lymphoma (16.3%) compared to multiple myeloma (MM) (36.8%), myeloproliferative malignancies (MPM) (54.6%), and chronic myeloid leukemia (CML) (72.2%) (Additional file 1: Fig. S1D). In solid cancers, the rates were lower in thoracic cancers (21.4%) and skin cancers (22.2%) compared to women’s cancers (47.4%), gastrointestinal (GI) cancers (50.0%), and urological cancers (66.7%) (Additional file 1: Fig. S1E). Subgroup analysis according to type of vaccine showed that the rate was higher with mRNA-1273 (80.4%) compared to BNT162b2 (53.0%) and AZD1222 (58.5%) (Additional file 1: Fig. S1F). Subgroup analysis according to type of therapy demonstrated that the rate was lowest with anti-CD20 therapy (10.0%) compared to chemotherapy (55.0%), kinase inhibitors (61.6%), immune check-point inhibitors (84.6%), or no therapy (71.5%) (Additional file 1: Fig. S1G).

### Rate of serologic response after two doses of COVID-19 vaccine

There were eleven studies (14 reports) that assessed the serologic response after two doses of COVID-19 vaccine [13, 14, 25–28, 30, 31, 33, 35, 36]. All studies did not delay the timing of the second dose including the one study reported from United Kingdom [25]. Three studies reported outcomes separately in hematologic and solid cancers [13, 14, 25]. As shown in Fig. 2B, the pooled proportion of patients achieving a serologic response was 87.7% (95% CI 82.5–91.5). Subgroup analysis demonstrated that the rate was numerically lower in hematologic cancers (63.7%) compared to solid cancers (94.9%). Surprisingly, the rates in patients with hematologic cancers after one and two doses were not considerably different (59.0–63.7%).

Heterogeneity was present (*I*^2^ = 94.2%) likely attributed to the difference in the reported rates between hematologic and solid cancers. Multivariate meta-regression was undertaken to further explore the cause of heterogeneity and demonstrated that the difference in study population (hematologic versus solid cancer) (coefficient − 2.16, 95% CI − 3.26 − (− 3.86), *P* < 0.001) was a significant source of heterogeneity (Additional file 1: Table S4).

Funnel plot of the studies included in the meta-analysis demonstrated no asymmetry, but publication bias was present by Egger’s (*P* = 0.014) but not Begg’s test (*P* = 0.27) (Additional file 1: Fig. S2A).

Sensitivity analysis was undertaken by excluding two preprint studies (Additional file 1: Fig. S2B). Exclusion of preprints demonstrated a serologic response rate similar to when they were included (87.7% vs. 91.6%). Remove one study analysis also showed that pooled rates were not markedly altered by any single study (Additional file 1: Fig. S2C). Excluding one study that included non-mRNA vaccines [13] demonstrated similar results (data not shown).

Similar to the results after one dose, subgroup analysis stratified by type of hematologic cancer demonstrated that rates were lower among conditions that mainly affect B-cells, such as CLL (41.9%), and lymphoma (52.4%), compared to MM (72.7%), AML/CML (75.0%), and MPM (88.0%) (Additional file 1: Fig. S2D). In solid cancers, the rates were slightly lower in women’s cancers (76.6%) and skin cancers (80.0%) compared to GI cancers (86.7%), urological cancers (87.5%), thoracic cancers (91.5%), and brain cancers (95.0%) (Additional file 1: Fig. S2E). Subgroup analysis according to type of vaccine showed that the rates were similar with mRNA-1273 (87.2%) and BNT162b2 (85.1%) (Additional file 1: Fig. S2F). Subgroup analysis according to type of therapy demonstrated that the rates were lower with chimeric antigen receptor (CAR) T-cell therapy (12.5%), anti-CD20 therapy (22.9%), kinase inhibitors (38.9%), daratumab (50.0%), and stem cell transplant (SCT) (73.1%) compared to chemotherapy (92.8%), protease inhibitors (92.9%), immune check-point inhibitors (95.2%), hormonal therapy (99.0%), or no therapy (82.3%) (Additional file 1: Fig. S2G).

### Comparison of serologic response after a single dose of COVID-19 vaccine to controls

**Fig. 3:**
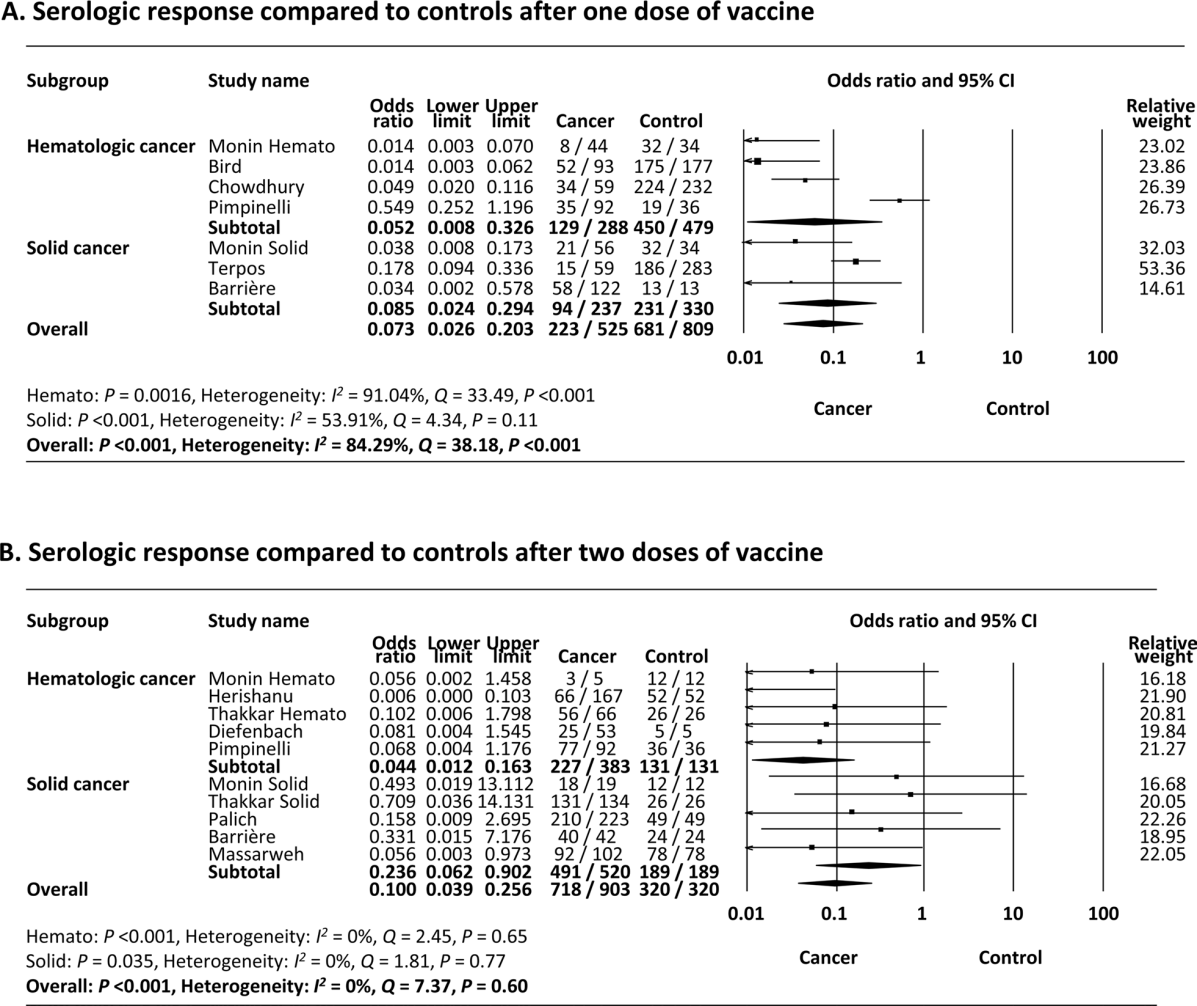
**A** Meta-analysis of serological response compared to controls after one dose of vaccine. **B** Meta-analysis of serological response compared to controls after two doses of vaccine

As shown in Fig. 3A, meta-analysis of 6 studies (7 reports) [25, 27, 29, 32–34] that included control patients demonstrated that a significantly lower proportion of cancer patients achieved a serologic response compared to control patients after a single dose of vaccine (odds ratio (OR) 0.073, 95% CI 0.026–0.20, *P* < 0.001). Subgroup analysis showed that both hematologic and solid cancers demonstrated lower response rates compared to controls (OR 0.052, 95% CI 0.008–0.33, *P* = 0.0016 and OR 0.085, 95% CI 0.024–0.29, *P* < 0.001, respectively).

Heterogeneity was present (*I*^2^ = 84.3%) likely attributed to the variable ratios reported among included studies (range 0.014–0.55), especially among studies included in hematologic cancers. Funnel plot of the studies included in the meta-analysis demonstrated no asymmetry (Begg’s *P* = 0.55, Egger’s *P* = 0.099) (Additional file 1: Fig. S3A).

### Comparison of serologic response after two doses of COVID-19 vaccine to controls

As shown in Fig. 3B, meta-analysis of 8 studies (10 reports) [13, 25–28, 31, 33, 36] that included control patients demonstrated that a significantly lower proportion of cancer patients achieved a serologic response compared to control patients after two doses of vaccine (OR 0.10 (95% CI 0.039–0.26), *P* < 0.001). Subgroup analysis showed that both hematologic and solid cancers demonstrated lower response rates compared to controls (OR 0.044, 95% CI 0.012–0.16, *P* < 0.001 and OR 0.24, 95% CI 0.062–0.90, *P* = 0.035, respectively), but the OR was much smaller in hematologic cancer.

Heterogeneity was not present (*I*^2^ = 0%) and funnel plot of the studies included in the meta-analysis demonstrated no asymmetry (Begg’s *P* = 0.15, Egger’s *P* = 0.15) (Additional file 1: Fig. S3B).

Some studies reported data of absolute values of antibody titers (Table 1). Cancer patients had values that were 1/3 to 1/10 compared to controls.

### Grading the quality of evidence

Based on the GRADE approach, an overall quality of evidence for this analysis was low as the data were obtained from observational studies and there were no specific factors to down- or up-grade the level of certainty (Additional file 1: Table S5).

## Discussion

In the present meta-analysis, we assessed the serologic response to COVID-19 vaccination in patients with cancer. We demonstrated that only 54% of patients with cancer achieved a serologic response to a single dose of COVID-19 vaccine, which improved to 88% after two doses. The rates were significantly lower compared to controls at both stages, especially in patients with hematologic cancers, suggesting the urgent need for an improved vaccination strategy in this vulnerable patient population. Further studies assessing the response in patients with various types of cancers or to other types of vaccine are warranted.

Patients with cancer are known to have a greater mortality due to COVID-19 [37]. Patients with cancer are immunocompromised due to the immunosuppressive properties of cancer and the effects of chemotherapy and radiation therapy [38]. Chemotherapy may cause long-term changes in immune parameters including B and T cell functions that increases the risk of various infections as well as hampers the response to vaccines [39]. They might also have a dysfunctional immune responses to infections secondary to immunotherapeutics, such as programmed cell death 1 or programmed cell death ligand 1 inhibitors, or chimeric antigen receptor (CAR)-modified T cell therapy [38]. Furthermore, patients with cancer are often older and carry comorbidities placing them at greater risk for morbidity and mortality due to COVID-19 [40].

Due to the lack of effective therapies to treat COVID-19, it is important to know the effectiveness of COVID-19 vaccines in patients with cancer. Roeker et al. reported that seroconversion of anti-SARS-CoV-2 antibody after two doses of COVID-19 vaccine was 52% in leukemia patients whereas Massarweh et al. reported a 90% seroconversion rate in solid cancer patients [28, 30]. A majority of studies have reported an attenuated response in patients with cancer, but the seroconversion rates varied largely and most studies were of small sample sizes. Therefore, it was important to integrate findings across studies to determine the serologic response rate to COVID-19 vaccination in patients with both hematologic and solid cancers.

Shroti et al. reported that nearly all patients developed antibodies after one or two doses of BNT162b2 or ChAdOx1 nCoV-19 vaccines (96.42% and 99.08%, respectively). However, they reported that elderly people and those with comorbidities such as diabetes, cardiovascular disease, and cancer had lower antibody levels [41]. Our study showed that the proportion of patients achieving a serologic response after a single or two doses of COVID-19 vaccine was 54% and 88%, respectively, which are much lower than the rates reported by Shroti et al. Among studies that included control patients without cancer, the OR of achieving serologic response among patients with cancer was significantly lower after the first and second dose. The results of our study are consistent with recent meta-analyses reporting lower response rates in hematological cancers vs. solid cancers or controls [42, 43].

The proportion of patients on anti-cancer therapy and older age were factors associated with lower vaccine response rates after the first dose whereas diagnosis of hematologic cancer was the only factor associated with lower response after the second dose. This supports the recommendations to prioritize cancer patients for additional measures such as booster vaccinations. We also analyzed vaccine response in different types of cancer as well as in patients receiving different treatments. Patients with CLL and lymphoma, which are mainly of B-cell origin, had lower serologic response rates compared to MM, AML/CML and MPM patients and therapies such as CAR-T therapy, anti-CD20 therapy, and kinase inhibitors were associated with lower rates. In contrast, most solid cancers had serologic response rates in 80–95% range after two doses of vaccine and therapies mostly used for these conditions such as chemotherapy, immune check-point inhibitors, and hormonal therapy had higher response rates.

### Limitations

Ten months have passed since the UK first approved BNT162b2, but available studies in cancer patients was still limited. There are currently 9 different vaccines on the global market, but nearly all of the included studies used only mRNA vaccines: either BNT162b2 (or mRNA-1273, and only a few studies included ChAdOx1 nCoV-19 or AD26.COV2.S in a small proportion of patients. Further research is needed whether the results of our study can be generalized to other types of vaccines. We assessed humoral responses to vaccination, but the extent to which cell-mediated immunity, such as spike-specific T cell response, is involved remains unclear. However, recent real-world studies have shown that antibody levels are associated/predictive of infection risk and that immunosuppressed patients were at risk for breakthrough infections [44, 45]. We undertook subgroup analyses according to different diseases or therapies, but the number of studies reporting detailed data were limited. Included studies mainly used one of the three commercially available antibody tests (Roche, DiaSorin, or Abbott), which all have excellent sensitivity (98–100%) [46]. As a result, they correlate well with each other in terms of seroprevalence. Some early studies delayed the timing of the second dose, but all of the studies included in our meta-analysis for the assessment following two doses administered the second dose without delay. Serologic response rates were similar after two doses of BNT162b2 or mRNA-1273 vaccines, but further studies are needed to assess the difference in degree of waning antibody levels in cancer patients [47]. Furthermore, we were not able to assess the vaccine effectiveness in preventing infections or hospitalizations in cancer patients.

## Conclusion

In the present comprehensive meta-analysis, we analyzed the rate of seroconversion to COVID-19 vaccines in patients with cancer. Our meta-analysis demonstrated that 54% and 88% of patients with cancer achieved a serologic response after one and two doses of COVID-19 vaccine, respectively, which were statistically lower compared to controls. Cancer patients should receive the series of two dose vaccines without delay and should continue to follow safety measures including mask-wearing after vaccination. Certain conditions and therapy were associated with lower response rates, so further studies assessing optimal prophylactic strategy in patients with cancer will be warranted.

## Supplementary Information

**Additional file 1. Supplementary Figure 1.** Funnel plot, sensitivity analyses, and subgroup analyses for serologic response after one dose of vaccine. **Supplementary Figure 2.** Funnel plot, sensitivity analyses, and subgroup analyses for serologic response after two doses of vaccine. **Supplementary Figure 3.** Funnel plots of studies included in meta-analyses of comparison of serologic response after one and two doses of vaccine compared to controls.

## Data Availability

Not applicable.
