# Supplementary material for: Serologic response following SARS-COV2 vaccination in patients with cancer: a systematic review and meta-analysis
Source: J Hematol Oncol. 2022 Feb 5;15:15. doi: 10.1186/s13045-022-01233-3 (PMC8817639; doi:10.1186/s13045-022-01233-3)
Supplement: Supplementary file 1 — Additional file 1. Supplementary Figure 1. Funnel plot, sensitivity analyses, and subgroup analyses for serologic response after one dose of vaccine. Supplementary Figure 2. Funnel plot, sensitivity analyses, and subgroup analyses for serologic response after two doses of vaccine. Supplementary Figure 3. Funnel plots of studies included in meta-analyses of comparison of serologic response after one and two doses of vaccine compared to controls. [file 13045_2022_1233_MOESM1_ESM.pdf]

Supplementary Figure 1.

A. Funnel plot of studies included in meta-analysis of serologic response after one dose of vaccine

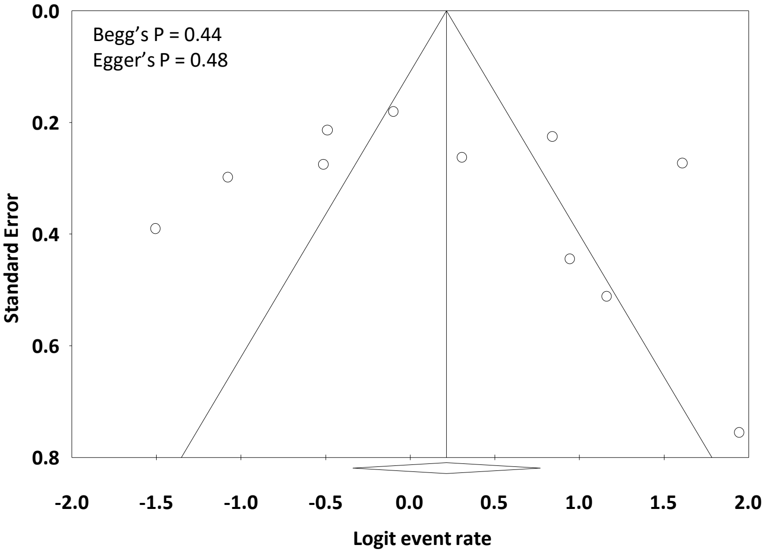

B. Sensitivity analysis excluding one study at a time for serologic response after one dose of vaccine

Removed study	Point	Lower limit	Upper limit
Monin Solid	0.555	0.416	0.686
Monin Hemato	0.574	0.447	0.691
Harrington CML	0.509	0.382	0.635
Bird	0.537	0.391	0.676
Chowdhury	0.534	0.393	0.670
Terpos	0.567	0.437	0.689
Harrington MPD	0.515	0.384	0.644
Pimpinelli	0.556	0.414	0.689
Addeo Solid	0.489	0.381	0.599
Addeo Hemato	0.519	0.386	0.649
Barrière	0.547	0.397	0.689
**Overall**	0.537	0.409	0.660

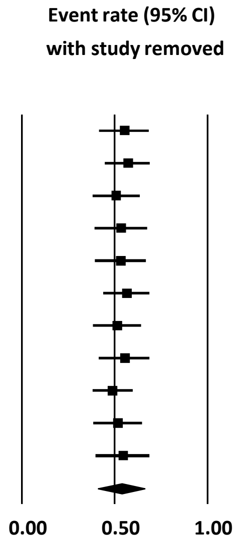

C. Sensitivity analysis excluding non-mRNA studies for serologic response after one dose of vaccine

Subgroup	Study name	Event rate	Lower limit	Upper limit	Total	Relative weight
**Hematologic cancer**	Monin Hemato	0.182	0.094	0.323	8 / 44	21.06
	Harrington CML	0.875	0.614	0.969	14 / 16	16.22
	Harrington MPD	0.762	0.540	0.897	16 / 21	19.53
	Pimpinelli	0.380	0.287	0.483	35 / 92	22.79
	Addeo Hemato	0.720	0.518	0.860	18 / 25	20.40
	**Subtotal**	**0.576**	**0.319**	**0.798**	**91 / 198**	
**Solid cancer**	Monin Solid	0.375	0.259	0.508	21 / 56	32.85
	Addeo Solid	0.833	0.745	0.895	80 / 96	32.89
	Barrière	0.475	0.388	0.564	58 / 122	34.26
	**Subtotal**	**0.581**	**0.304**	**0.815**	**159 / 274**	
**Overall**		**0.579**	**0.385**	**0.750**	**250 / 472**	

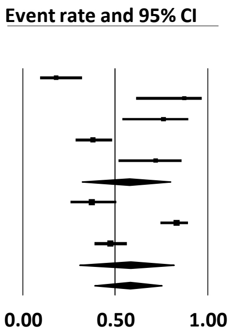

Hemato: Heterogeneity: I^2 = 88.46%, Q = 34.67, P <0.001

Solid: Heterogeneity: I^2 = 94.41%, Q = 35.83, P <0.001

Overall: Heterogeneity: I^2 = 90.58%, Q = 74.37, P <0.001

Supplementary Figure 1.

D. Subgroup analysis stratified by type of disease in hematologic cancer studies for serologic response after one dose of vaccine

Disease	Study	Event rate	Lower limit	Upper limit	Total	Relative weight
**CLL**	Monin Hemato CLL	0.167	0.023	0.631	1 / 6	100.00
**Subtotal**		**0.167**	**0.023**	**0.631**	**1 / 6**	
**CML**	Monin Hemato CML	0.167	0.010	0.806	0 / 2	17.55
	Harrington CML	0.875	0.614	0.969	14 / 16	39.37
	Chowdhury CML	0.750	0.448	0.917	9 / 12	43.08
**Subtotal**		**0.722**	**0.369**	**0.920**	**23 / 30**	
**Lymphoma**	Monin Hemato Lymphoma	0.133	0.034	0.405	2 / 15	69.80
	Monin Hemato ALCL	0.250	0.034	0.762	1 / 4	30.20
**Subtotal**		**0.163**	**0.053**	**0.403**	**3 / 19**	
**Mix**	Addeo Hemato	0.720	0.518	0.860	18 / 25	100.00
**Subtotal**		**0.720**	**0.518**	**0.860**	**18 / 25**	
**MM**	Monin Hemato MM	0.333	0.111	0.667	3 / 9	25.63
	Bird MM	0.559	0.457	0.656	52 / 93	39.18
	Pimpinelli MM	0.214	0.115	0.363	9 / 42	35.19
**Subtotal**		**0.368**	**0.157**	**0.645**	**64 / 144**	
**MPN**	Monin Hemato MDS/MPN	0.500	0.059	0.941	1 / 2	1.83
	Chowdhury ET	0.647	0.404	0.832	11 / 17	14.24
	Chowdhury PV	0.455	0.203	0.732	5 / 11	10.01
	Chowdhury MF	0.571	0.230	0.856	4 / 7	6.29
	Chowdhury MDS	0.462	0.224	0.718	6 / 13	11.85
	Harrington ET	0.944	0.495	0.997	8 / 8	1.73
	Harrington MF	0.750	0.377	0.937	6 / 8	5.50
	Harrington PV	0.250	0.034	0.762	1 / 4	2.75
	Pimpinelli MPN	0.520	0.383	0.654	26 / 50	45.79
**Subtotal**		**0.546**	**0.452**	**0.636**	**68 / 120**	
**Overall**		**0.527**	**0.450**	**0.603**	**177 / 344**	

Event rate and 95% CI

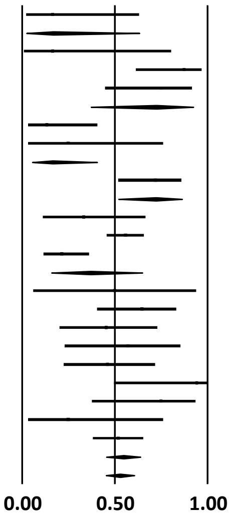

CLL: Heterogeneity: I^2 = 0%, Q = 0, P = 1.00

CML: Heterogeneity: I^2 = 53.31%, Q = 4.28, P = 0.12

Lymphoma: Heterogeneity: I^2 = 0%, Q = 0.31, P = 0.58

Mix: I^2 = 0%, Q = 0, P = 1.00

MM: I^2 = 85.04%, Q = 13.37, P = 0.001

MPN: I^2 = 0%, Q = 7.40, P = 0.49

Overall: Heterogeneity: I^2 = 61.12%, Q = 46.30, P <0.001

E. Subgroup analysis stratified by type of disease in solid organ cancer studies for serologic response after one dose of vaccine

Disease	Study	Event rate	Lower limit	Upper limit	Total	Relative weight
**GI cancers**	Monin Solid GI	0.500	0.168	0.832	3 / 6	100.00
**Subtotal**		**0.500**	**0.168**	**0.832**	**3 / 6**	
**Mix**	Terpos Mix	0.254	0.159	0.380	15 / 59	32.79
	Addeo Solid	0.833	0.745	0.895	80 / 96	33.11
	Barrière	0.475	0.388	0.564	58 / 122	34.10
**Subtotal**		**0.537**	**0.229**	**0.819**	**153 / 277**	
**Skin cancers**	Monin Solid Skin	0.222	0.056	0.579	2 / 9	100.00
**Subtotal**		**0.222**	**0.056**	**0.579**	**2 / 9**	
**Thoracic cancers**	Monin Solid Thoracic	0.214	0.071	0.494	3 / 14	100.00
**Subtotal**		**0.214**	**0.071**	**0.494**	**3 / 14**	
**Urological cancers**	Monin Solid Uro	0.667	0.268	0.916	4 / 6	100.00
**Subtotal**		**0.667**	**0.268**	**0.916**	**4 / 6**	
**Women's cancers**	Monin Solid Women's	0.474	0.268	0.689	9 / 19	100.00
**Subtotal**		**0.474**	**0.268**	**0.689**	**9 / 19**	
**Overall**		**0.422**	**0.300**	**0.554**	**174 / 331**	

Event rate and 95% CI

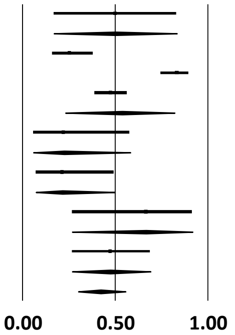

GI: Heterogeneity: I^2 = 0%, Q = 0, P = 1.00

Mix: Heterogeneity: I^2 = 95.75%, Q = 47.04, P <0.001

Skin: Heterogeneity: I^2 = 0%, Q = 0, P = 1.00

Thoracic : Heterogeneity: I^2 = 0%, Q = 0, P = 1.00

Uro: Heterogeneity: I^2 = 0%, Q = 0, P = 1.00

Women's: Heterogeneity : I^2 = 0%, Q = 0, P = 1.00

Overall: Heterogeneity: I^2 = 87.24%, Q = 54.85, P <0.001

Supplementary Figure 1.

F. Subgroup analysis stratified by type of vaccine in hematologic and solid organ cancer studies for serologic response after one dose of vaccine

Vaccine type	Study	Type of cancer	Event rate	Lower limit	Upper limit	Total	Relative weight
**AZD1222**	Bird AZD1222	Hematologic cancer	0.578	0.431	0.712	26 / 45	55.17
	Chowdhury AZD1222	Hematologic cancer	0.595	0.432	0.739	22 / 37	44.83
**Subtotal**			**0.585**	**0.476**	**0.687**	**48 / 82**	
**BNT162b2**	Monin Solid	Solid cancer	0.375	0.259	0.508	21 / 56	12.72
	Monin Hemato	Hematologic cancer	0.182	0.094	0.323	8 / 44	11.12
	Harrington CML	Hematologic cancer	0.875	0.614	0.969	14 / 16	6.58
	Bird BNT162b2	Hematologic cancer	0.542	0.401	0.676	26 / 48	12.54
	Chowdhury BNT162b2	Hematologic cancer	0.545	0.341	0.735	12 / 22	10.58
	Harrington MPD BNT162b2	Hematologic cancer	0.762	0.540	0.897	16 / 21	9.41
	Pimpinelli BNT162b2	Hematologic cancer	0.380	0.287	0.483	35 / 92	13.49
	Addeo BNT162b2	Solid/Hemato	0.828	0.647	0.926	24 / 29	9.70
	Barrière BNT162b2	Solid cancer	0.475	0.388	0.564	58 / 122	13.86
**Subtotal**			**0.530**	**0.404**	**0.652**	**214 / 450**	
**mRNA-1273**	Addeo mRNA-1273	Solid/Hemato	0.804	0.711	0.873	74 / 92	100.00
**Subtotal**			**0.804**	**0.711**	**0.873**	**74 / 92**	
**Overall**			**0.644**	**0.577**	**0.705**	**336 / 624**	

Event rate and 95% CI

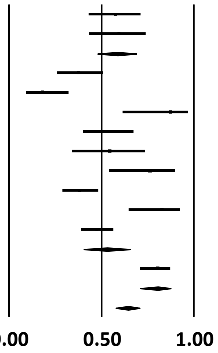

AZD1222: Heterogeneity: I^2 = 0%, Q = 0.024, P = 0.88

BNT162b2: Heterogeneity: I^2 = 82.02%, Q = 44.50, P <0.001

mRNA-1273: Heterogeneity: I^2 = 0%, Q = 0, P = 1.00

Overall: Heterogeneity: I^2 = 85.51%, Q = 75.90, P <0.001

G. Subgroup analysis stratified by type of therapy in hematologic and solid organ cancer studies for serologic response after one dose of vaccine

Therapy	Study	Type of cancer	Event rate	Lower limit	Upper limit	Total	Relative weight
**Anti-CD20**	Addeo Anti-CD20	Hemato/Solid	0.100	0.006	0.674	0 / 4	100.00
**Subtotal**			**0.100**	**0.006**	**0.674**	**0 / 4**	
**Chemotherapy**	Addeo Chemotherapy	Hemato/Solid	0.690	0.503	0.830	20 / 29	44.82
	Barrière Chemotherapy	Solid cancer	0.429	0.338	0.525	45 / 105	55.18
**Subtotal**			**0.550**	**0.297**	**0.779**	**65 / 134**	
**Hydroxycarbamide**	Chowdhury Hydroxycarbamide	Hematologic cancer	0.364	0.143	0.661	4 / 11	62.04
	Harrington Hydroxycarbamide	Hematologic cancer	0.900	0.326	0.994	4 / 4	37.96
**Subtotal**			**0.619**	**0.106**	**0.957**	**8 / 15**	
**Kinase inhibitors**	Chowdhury Ruxolitinb	Hematologic cancer	0.100	0.006	0.674	0 / 4	23.73
	Harrington Ruxolitinib	Hematologic cancer	0.667	0.268	0.916	4 / 6	36.85
	Addeo Kinase inhibitors	Hemato/Solid	0.867	0.595	0.966	13 / 15	39.42
**Subtotal**			**0.616**	**0.187**	**0.918**	**17 / 25**	
**ICI**	Addeo ICI	Hemato/Solid	0.846	0.549	0.961	11 / 13	100.00
**Subtotal**			**0.846**	**0.549**	**0.961**	**11 / 13**	
**IFN**	Chowdhury IFN	Hematologic cancer	0.875	0.463	0.983	7 / 8	66.04
	Harrington IFN	Hematologic cancer	0.900	0.326	0.994	4 / 4	33.96
**Subtotal**			**0.884**	**0.581**	**0.977**	**11 / 12**	
**Targeted therapy**	Barrière Targeted therapy	Solid cancer	0.765	0.514	0.909	13 / 17	100.00
**Subtotal**			**0.765**	**0.514**	**0.909**	**13 / 17**	
**No therapy**	Chowdhury No therapy	Hematologic cancer	0.583	0.308	0.815	7 / 12	33.51
	Harrington No therapy	Hematologic cancer	0.571	0.230	0.856	4 / 7	26.86
	Addeo No therapy	Hemato/Solid	0.864	0.728	0.937	38 / 44	39.63
**Subtotal**			**0.715**	**0.451**	**0.885**	**49 / 63**	
**Overall**			**0.701**	**0.584**	**0.797**	**174 / 283**	

Event rate and 95% CI

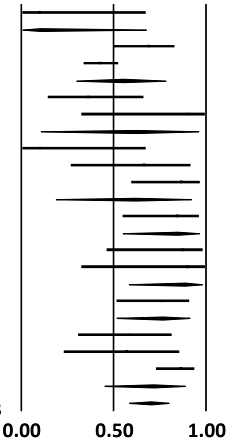

Anti-CD20: Heterogeneity: I^2 = 0%, Q = 0, P = 1.00

Chemotherapy: Heterogeneity: I^2 = 83.05%, Q = 5.90, P = 0.015

Hydroxycarbamide : Heterogeneity: I^2 = 65.59%, Q = 2.91, P = 0.088

Kinase inhibitors: I^2 = 66.73%, Q = 6.01, P = 0.050

ICI: I^2 = 0%, Q = 0, P = 1.00

IFN: I^2 = 0%, Q = 0.019, P = 0.89

Targeted therapy: I^2 = 0%, Q = 0, P = 1.00

No therapy: I^2 = 64.96%, Q = 5.71, P = 0.058

Overall: Heterogeneity: I^2 = 69.52%, Q = 45.93, P <0.001

Supplementary Figure 2.

A. Funnel plot of studies included in meta-analysis of serologic response after two doses of vaccine

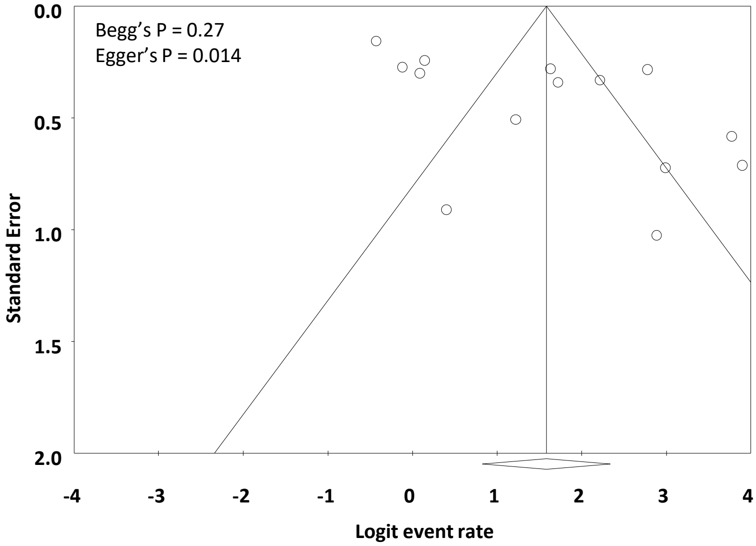

B. Sensitivity analysis excluding preprints for serologic response after two doses of vaccine

Subgroup	Study name	Event rate	Lower limit	Upper limit	Total	Relative weight
**Hematologic cancer**	Monin Hemato	0.600	0.200	0.900	3 / 5	11.28
	Herishanu	0.395	0.324	0.471	66 / 167	19.02
	Roeker	0.523	0.377	0.664	23 / 44	18.01
	Thakkar Hemato	0.848	0.741	0.916	56 / 66	17.63
	Pimpinelli	0.837	0.747	0.899	77 / 92	18.18
	Addeo Hemato	0.773	0.556	0.902	17 / 22	15.87
	**Subtotal**	**0.685**	**0.462**	**0.846**	**242 / 396**	
**Solid cancer**	Monin Solid	0.947	0.706	0.993	18 / 19	6.07
	Thakkar Solid	0.978	0.933	0.993	131 / 134	14.70
	Palich	0.942	0.902	0.966	210 / 223	30.36
	Addeo Solid	0.980	0.924	0.995	99 / 101	11.00
	Barrière	0.952	0.829	0.988	40 / 42	10.76
	Massarweh	0.902	0.827	0.946	92 / 102	27.11
	**Subtotal**	**0.949**	**0.916**	**0.970**	**590 / 621**	
**Overall**		**0.916**	**0.874**	**0.946**	**832 / 1017**	

Event rate and 95% CI

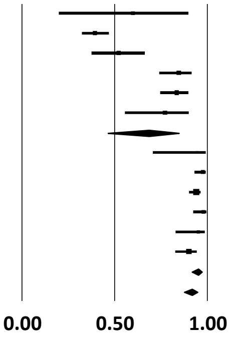

Hemato: Heterogeneity: I^2 = 92.23%, Q = 64.35, P <0.001

Solid: Heterogeneity: I^2 = 39.79%, Q = 8.31, P = 0.14

Overall: Heterogeneity: I^2 = 94.57%, Q = 202.68, P <0.001

C. Sensitivity analysis excluding one study at a time for serologic response after two doses of vaccine

Removed study	Point	Lower limit	Upper limit
Monin Solid	0.82	0.68	0.91
Monin Hemato	0.84	0.70	0.92
Herishanu	0.85	0.73	0.92
Agha	0.85	0.71	0.93
Roeker	0.85	0.71	0.93
Thakkar Solid	0.80	0.66	0.90
Thakkar Hemato	0.83	0.68	0.91
Palich	0.81	0.68	0.90
Diefenbach	0.85	0.71	0.93
Pimpinelli	0.83	0.68	0.92
Addeo Solid	0.81	0.66	0.90
Addeo Hemato	0.83	0.69	0.92
Barrière	0.82	0.67	0.91
Massarweh	0.82	0.68	0.91
**Overall**	0.83	0.70	0.91

Event rate (95% CI) with study removed

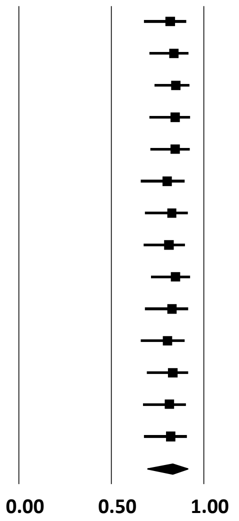

Supplementary Figure 2.

D. Subgroup analysis stratified by type of disease in hematologic cancer studies for serologic response after two doses of vaccine

Disease	Study	Event rate	Lower limit	Upper limit	Total	Relative weight
**CLL**	Monin CLL	0.500	0.059	0.941	1 / 2	2.14
	Herishanu CLL	0.395	0.324	0.471	66 / 167	57.82
	Agha CLL	0.231	0.076	0.522	3 / 13	9.08
	Roeker CLL	0.523	0.377	0.664	23 / 44	30.95
**Subtotal**		**0.419**	**0.323**	**0.521**	**93 / 226**	
**Lymphoma**	Agha Lymphoma	0.524	0.318	0.721	11 / 21	100.00
**Subtotal**		**0.524**	**0.318**	**0.721**	**11 / 21**	
**MM**	Agha MM	0.655	0.469	0.803	19 / 29	48.70
	Pimpinelli MM	0.786	0.637	0.885	33 / 42	51.30
**Subtotal**		**0.727**	**0.583**	**0.835**	**52 / 71**	
**AML/CML**	Agha AML/CML	0.750	0.238	0.966	3 / 4	100.00
**Subtotal**		**0.750**	**0.238**	**0.966**	**3 / 4**	
**MPN**	Pimpinelli MPN	0.880	0.758	0.945	44 / 50	100.00
**Subtotal**		**0.880**	**0.758**	**0.945**	**44 / 50**	
**Overall**		**0.577**	**0.503**	**0.648**	**203 / 372**	

Event rate and 95% CI

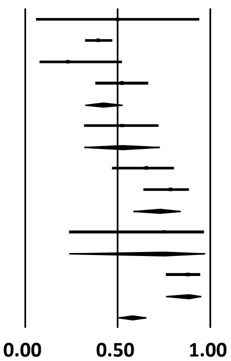

CLL: Heterogeneity: I^2 = 26.94%, Q = 4.11, P = 0.25

Lymphoma: Heterogeneity: I^2 = 0%, Q = 0, P = 1.00

MM: I^2 = 31.97%, Q = 1.47, P = 0.23

AML/CML: Heterogeneity: I^2 = 0%, Q = 0, P = 1.00

MPN: I^2 = 0%, Q = 0, P = 1.00

Overall: Heterogeneity: I^2 = 82.86%, Q = 46.68, P <0.001

E. Subgroup analysis stratified by type of disease in solid organ cancer studies for serologic response after two doses of vaccine

Disease	Study	Event rate	Lower limit	Upper limit	Total	Relative weight
**Brain**	Massarweh Brain	0.950	0.525	0.997	9 / 9	100.00
**Subtotal**		**0.950**	**0.525**	**0.997**	**9 / 9**	
**GI**	Monin GI	0.900	0.326	0.994	4 / 4	11.54
	Massarweh GI	0.862	0.685	0.947	25 / 29	88.46
**Subtotal**		**0.867**	**0.707**	**0.946**	**29 / 33**	
**Skin**	Monin Skin	0.800	0.309	0.973	4 / 5	100.00
**Subtotal**		**0.800**	**0.309**	**0.973**	**4 / 5**	
**Thoracic**	Monin Thoracic	0.875	0.266	0.993	3 / 3	19.16
	Massarweh Thoracic	0.923	0.739	0.981	24 / 26	80.84
**Subtotal**		**0.915**	**0.747**	**0.975**	**27 / 29**	
**Urological**	Massarweh Uro	0.875	0.463	0.983	7 / 8	100.00
**Subtotal**		**0.875**	**0.463**	**0.983**	**7 / 8**	
**Women's**	Monin Women's	0.929	0.423	0.996	6 / 6	26.40
	Massarweh Women's	0.667	0.333	0.889	6 / 9	73.60
**Subtotal**		**0.766**	**0.394**	**0.943**	**12 / 15**	
**Overall**		**0.871**	**0.783**	**0.927**	**88 / 99**	

Event rate and 95% CI

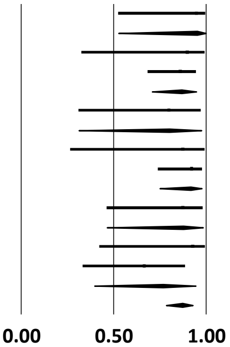

Brain: Heterogeneity: I^2 = 0%, Q = 0, P = 1.00

GI: Heterogeneity: I^2 = 0%, Q = 0.053, P = 0.82

Skin: Heterogeneity: I^2 = 0%, Q = 0, P = 1.00

Thoracic : Heterogeneity: I^2 = 0%, Q = 0.10, P = 0.75

Urological: Heterogeneity: I^2 = 0%, Q = 0, P = 1.00

Women's: Heterogeneity : I^2 = 24.26%, Q = 1.32, P = 0.25

Overall: Heterogeneity: I^2 = 0%, Q = 4.45, P = 0.82

Supplementary Figure 2.

F. Subgroup analysis stratified by type of vaccine in hematologic and solid organ cancer studies for serologic response after two dose of vaccine

Vaccine type	Study	Type of cancer	Event rate	Lower limit	Upper limit	Total	Relative weight
**BNT162b2**	Monin Solid	Solid cancer	0.947	0.706	0.993	18 / 19	7.92
	Monin Hemato	Hematologic cancer	0.600	0.200	0.900	3 / 5	8.45
	Herishanu	Hematologic cancer	0.395	0.324	0.471	66 / 167	11.15
	Agha BNT162b2	Hematologic cancer	0.441	0.286	0.608	15 / 34	10.75
	Thakkar BNT162b2	Hemato/Solid cancer	0.948	0.889	0.976	109 / 115	10.52
	Palich	Solid cancer	0.942	0.902	0.966	210 / 223	10.91
	Pimpinelli	Hematologic cancer	0.837	0.747	0.899	77 / 92	10.92
	Addeo BNT162b2	Hemato/Solid cancer	0.933	0.769	0.983	28 / 30	9.28
	Barrière	Solid cancer	0.952	0.829	0.988	40 / 42	9.31
	Massarweh	Solid cancer	0.902	0.827	0.946	92 / 102	10.79
**Subtotal**			**0.851**	**0.668**	**0.942**	**658 / 829**	
**Mix**	Roeker	Hematologic cancer	0.523	0.377	0.664	23 / 44	45.39
	Diefenbach	Hematologic cancer	0.472	0.342	0.605	25 / 53	54.61
**Subtotal**			**0.495**	**0.397**	**0.593**	**48 / 97**	
**mRNA-1273**	Agha mRNA-1273	Hematologic cancer	0.571	0.387	0.738	16 / 28	34.21
	Thakkar mRNA-1273	Hemato/Solid cancer	0.935	0.840	0.976	58 / 62	32.52
	Addeo mRNA-1273	Hemato/Solid cancer	0.946	0.877	0.977	88 / 93	33.27
**Subtotal**			**0.872**	**0.542**	**0.975**	**162 / 183**	
**Overall**			**0.569**	**0.479**	**0.655**	**868 / 1109**	

Event rate and 95% CI

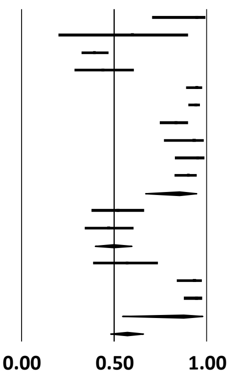

BNT162b2: Heterogeneity: I^2 = 95.20%, Q = 187.58, P <0.001

Mix: Heterogeneity: I^2 = 0%, Q = 0.25, P = 0.62

mRNA-1273: Heterogeneity: I^2 = 91.55%, Q = 233.67, P <0.001

Overall: Heterogeneity: I^2 = 94.18%, Q = 240.44, P <0.001

G. Subgroup analysis stratified by type of therapy in hematologic and solid organ cancer studies for serologic response after two doses of vaccine

Therapy	Study	Type of cancer	Event rate	Lower limit	Upper limit	Total	Relative weight
**Anti-CD20**	Herishanu Venetoclax/CD20	Hematologic cancer	0.136	0.045	0.348	3 / 22	19.55
	Roeker CD20	Hematologic cancer	0.143	0.036	0.427	2 / 14	17.62
	Roeker Venetoclax/CD20	Hematologic cancer	0.063	0.004	0.539	0 / 7	9.91
	Thakkar CD20	Hematologic cancer	0.696	0.485	0.847	16 / 23	21.74
	Diefenbach CD20	Hematologic cancer	0.250	0.117	0.456	6 / 24	21.51
	Addeo CD20	Hematologic cancer	0.100	0.006	0.674	0 / 4	9.67
**Subtotal**			**0.229**	**0.085**	**0.487**	**27 / 94**	
**Any active therapy**	Agha Active tx	Hematologic cancer	0.500	0.328	0.672	15 / 30	100.00
**Subtotal**			**0.500**	**0.328**	**0.672**	**15 / 30**	
**CAR-T**	Thakkar CAR-T	Hematologic cancer	0.125	0.007	0.734	0 / 3	100.00
**Subtotal**			**0.125**	**0.007**	**0.734**	**0 / 3**	
**Chemotherapy**	Diefenbach Chemotx	Hematologic cancer	0.900	0.326	0.994	4 / 4	19.42
	Addeo Chemotx	Hemato/Solid cancer	0.933	0.769	0.983	28 / 30	80.58
**Subtotal**			**0.928**	**0.780**	**0.979**	**32 / 34**	
**Daratumab**	Pimpinelli	Hematologic cancer	0.500	0.260	0.740	7 / 14	100.00
**Subtotal**			**0.500**	**0.260**	**0.740**	**7 / 14**	
**Hornmonal therapy**	Thakkar Hormonal tx	Solid cancer	0.990	0.854	0.999	47 / 47	100.00
**Subtotal**			**0.990**	**0.854**	**0.999**	**47 / 47**	
**ICI**	Thakkar ICI	Hemato/Solid cancer	0.968	0.804	0.995	30 / 31	51.03
	Addeo ICI	Hemato/Solid cancer	0.929	0.630	0.990	13 / 14	48.97
**Subtotal**			**0.952**	**0.828**	**0.988**	**43 / 45**	
**Kinase inhibitors**	Herishanu BTK inh	Hematologic cancer	0.160	0.082	0.289	8 / 50	29.65
	Roeker BTK inh	Hematologic cancer	0.214	0.071	0.494	3 / 14	25.54
	Diefenbach BTK inh	Hematologic cancer	0.400	0.158	0.703	4 / 10	25.64
	Addeo Kinase inh	Hematologic cancer	0.923	0.609	0.989	12 / 13	19.18
**Subtotal**			**0.389**	**0.136**	**0.720**	**27 / 87**	
**No therapy**	Herishanu No tx	Hematologic cancer	0.587	0.484	0.683	54 / 92	21.57
	Agha No tx	Hematologic cancer	0.568	0.406	0.716	21 / 37	20.39
	Roeker No tx	Hematologic cancer	0.944	0.693	0.992	17 / 18	11.37
	Thakkar No tx	Hemato/Solid cancer	0.932	0.833	0.974	55 / 59	18.01
	Diefenbach No tx	Hematologic cancer	0.733	0.467	0.896	11 / 15	17.09
	Addeo No tx	Hemato/Solid cancer	0.978	0.858	0.997	44 / 45	11.56
**Subtotal**			**0.823**	**0.638**	**0.924**	**202 / 266**	
**Proteasome inhibitors**	Pimpinelli PI/IMiD	Hematologic cancer	0.929	0.755	0.982	26 / 28	100.00
**Subtotal**			**0.929**	**0.755**	**0.982**	**26 / 28**	
**SCT**	ThakkarSCT	Hematologic cancer	0.731	0.533	0.866	19 / 26	100.00
**Subtotal**			**0.731**	**0.533**	**0.866**	**19 / 26**	
**Overall**			**0.685**	**0.605**	**0.754**	**445 / 674**	

Event rate and 95% CI

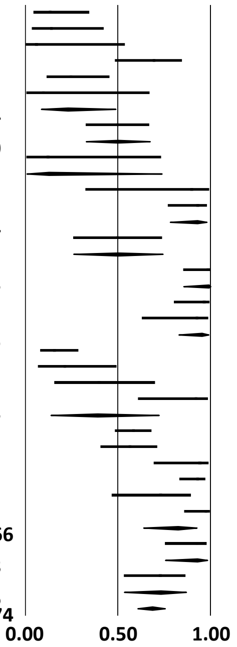

Anti-CD20: Heterogeneity: I^2 = 75.45%, Q = 20.36, P = 0.001

Any active therapy: Heterogeneity: I^2 = 0%, Q = 0, P = 1.00

CAR-T: Heterogeneity: I^2 = 0%, Q = 0, P = 1.00

Chemotherapy: Heterogeneity: I^2 = 0%, Q = 0.071, P = 0.89

Daratumumab: Heterogeneity: I^2 = 0%, Q = 0, P = 1.00

Hormonal therapy : Heterogeneity: I^2 = 0%, Q = 0, P = 1.00

ICI: I^2 = 0%, Q = 0.33, P = 0.57

Kinase inhibitors: I^2 = 80.21%, Q = 15.16, P = 0.002

No therapy: I^2 = 84.36%, Q = 31.97, P <0.001

Proteasome inhibitors/IMiD: I^2 = 0%, Q = 0, P = 1.00

SCT: I^2 = 0%, Q = 0, P = 1.00

Overall: Heterogeneity: I^2 = 84.72%, Q = 163.66, P <0.001

Supplementary Figure 3.

A. Funnel plot of studies included in meta-analysis of comparison of serologic response after one dose of vaccine compared to controls

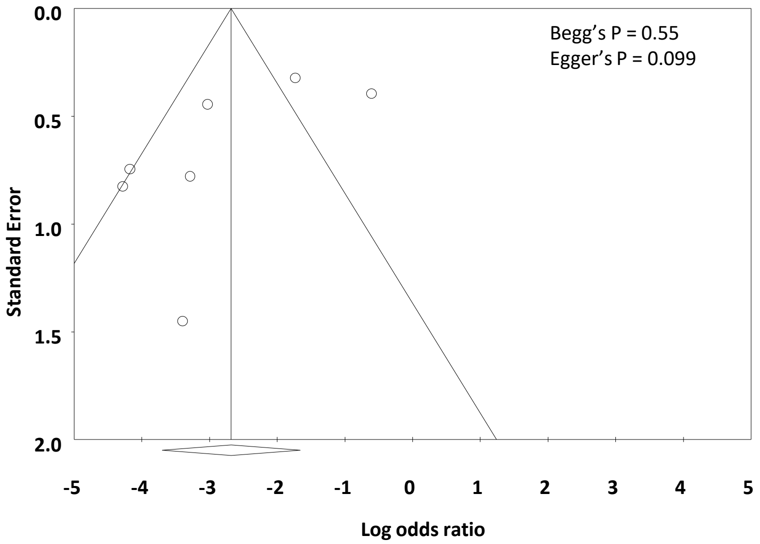

B. Funnel plot of studies included in meta-analysis of comparison of serologic response after two doses of vaccine compared to controls

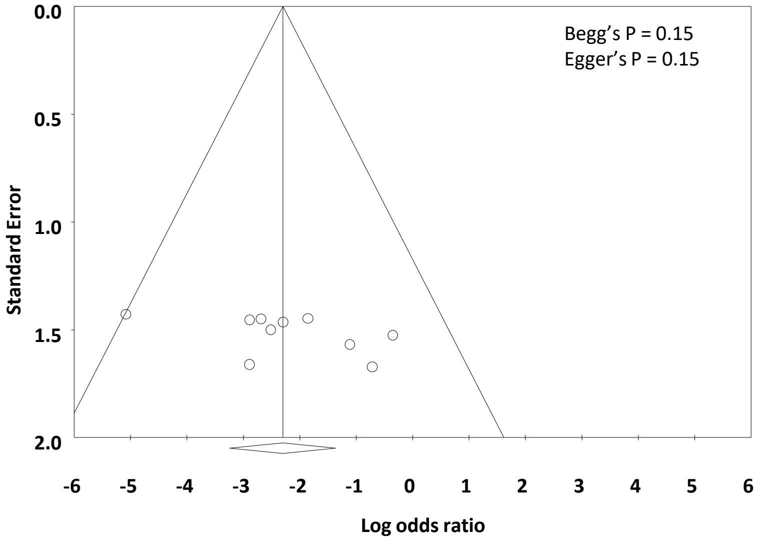

Supplementary Figure Legends

Supplementary Figure 1. (A) Funnel plot of studies included in meta-analysis of serological response after one dose of vaccine. Begg's test $P = 0.44$; Egger's tests $P = 0.48$. **(B)** Sensitivity analysis excluding one study at a time for serologic response after one dose of vaccine. **(C)** Sensitivity analysis excluding non-mRNA studies for serologic response after one dose of vaccine. **(D)** Subgroup analysis stratified by type of disease in hematologic cancer studies for serologic response after one dose of vaccine. **(E)** Subgroup analysis stratified by type of disease in solid organ cancer studies for serologic response after one dose of vaccine. **(F)** Subgroup analysis stratified by type of vaccine in hematologic and solid organ cancer studies for serologic response after one dose of vaccine. **(G)** Subgroup analysis stratified by type of therapy in hematologic and solid organ cancer studies for serologic response after one dose of vaccine.

Supplementary Figure 2. (A) Funnel plot of studies included in meta-analysis of serological response after two doses of vaccine. Begg's test $P = 0.27$; Egger's tests $P = 0.014$. **(B)** Sensitivity analysis excluding preprints for serologic response after two doses of vaccine. **(C)** Sensitivity analysis excluding one study at a time for serologic response after two doses of vaccine. **(D)** Subgroup analysis stratified by type of disease in hematologic cancer studies for serologic response after two doses of vaccine. **(E)** Subgroup analysis stratified by type of disease in solid organ cancer studies for serologic response after two doses of vaccine. **(F)** Subgroup analysis stratified by type of vaccine in hematologic and solid organ cancer studies for serologic response after two doses of vaccine. **(G)** Subgroup analysis stratified by type of therapy in hematologic and solid organ cancer studies for serologic response after two doses of vaccine.

Supplementary Figure 3. (A) Funnel plot of studies included in meta-analysis of comparison of serological response after one dose of vaccine compared to controls. Begg's test $P = 0.55$; Egger's tests $P = 0.099$. **(B)** Funnel plot of studies included in meta-analysis of comparison of serological response after two doses of vaccine compared to controls. Begg's test $P = 0.15$; Egger's tests $P = 0.15$.

Supplementary Table 1. Search strategy

**PubMed search strategy**		**Number of studies**
#1	"COVID-19" OR "SARS-CoV-2" [MeSH Terms]	98,051
#2	"cancer" OR "malignancy" OR "leukemia" OR "lymphoma" OR "immunosuppressed" OR "hematologic diseases" [MeSH Terms]	2,567,229
#3	"vaccine" OR "immunization" [MeSH Terms]	162,867
#4	#1 AND #2 AND #3	602
**EMBASE search strategy**		**Number of studies**
#1	"COVID-19" OR "SARS-CoV-2" [MeSH Terms]	87,226
#2	"cancer" OR "malignancy" OR "leukemia" OR "lymphoma" OR "immunosuppressed" OR "hematologic diseases" [MeSH Terms]	2,015,921
#3	"vaccine" OR "immunization" [MeSH Terms]	139,516
#4	#1 AND #2 AND #3	591
**medRxiv search strategy**		**Number of studies**
#1	"COVID-19" OR "SARS-CoV-2"	14,517
#2	"cancer" OR "malignancy" OR "leukemia" OR "lymphoma" OR "immunosuppressed" OR "hematologic diseases"	21,185
#3	"vaccine" OR "immunization"	11,642
#4	#1 AND #2 AND #3	420

Supplementary Table 2. Risk of bias assessment by Joanna Briggs Institute Critical Appraisal Checklist

Author	Year	Q1	Q2	Q3	Q4	Q5	Q6	Q7	Q8	Q9
**Monin**	2021	Yes	Yes	Yes	Yes	Yes	Yes	Yes	Yes	Yes
**Harrington**	2021	Yes	Yes	Yes	No	Yes	Yes	Yes	Yes	Yes
**Bird**	2021	Yes	Yes	Yes	Unclear	No	NA	Yes	Yes	Yes
**Herishanu**	2021	Yes	Yes	Yes	Yes	No	Yes	Yes	Yes	Yes
**Agha**	2021	Yes	Yes	Yes	No	No	Yes	Yes	Yes	Yes
**Roeker**	2021	Yes	Yes	Yes	No	Yes	Yes	Yes	Yes	Yes
**Thakkar**	2021	Yes	Yes	Yes	Yes	No	Yes	Yes	Yes	Yes
**Palich**	2021	Yes	Yes	Yes	Yes	Yes	Yes	No	Yes	Yes
**Chowdhury**	2021	Yes	Yes	Yes	Yes	No	Yes	Unclear	Yes	Yes
**Diefenbach**	2021	Yes	Yes	Yes	Yes	Unclear	Unclear	Yes	Yes	Unclear
**Terpos**	2021	Yes	Yes	Yes	Yes	Yes	Yes	Yes	Yes	Yes
**Harrington**	2021	Unclear	Yes	Yes	No	Yes	Yes	Yes	Yes	Yes
**Pimpinelli**	2021	Yes	Yes	Yes	Yes	Yes	Yes	Yes	Yes	Yes
**Addeo**	2021	Yes	Yes	Yes	No	Yes	No	Yes	Yes	Yes
**Barrière**	2021	Yes	Yes	Yes	Yes	Yes	No	Yes	Yes	Yes
**Massarweh**	2021	Yes	Yes	Yes	Yes	No	Yes	Yes	Yes	Yes

Q1: Is it clear in the study what is the 'cause' and what is the 'effect' (i.e. there is no confusion about which variable comes first)?; Q2: Were the participants included in any comparisons similar?; Q3: Were the participants included in any comparisons receiving similar treatment/care, other than the exposure or intervention of interest?; Q4: Was there a control group?; Q5: Were there multiple measurements of the outcome both pre and post the intervention/exposure?; Q6: Was follow up complete and if not, were differences between groups in terms of their follow up adequately described and analyzed?; Q7: Were the outcomes of participants included in any comparisons measured in the same way?; Q8: Were outcomes measured in a reliable way?; Q9: Was appropriate statistical analysis used?; NA not applicable.

Supplementary Table 3. Univariate and multivariate meta-regression models of variables associated with serologic response after one dose of vaccine

	Univariate meta-regression			Multivariate meta-regression		
	**Coefficient**	**95% CI**	**P-value**	**Coefficient**	**95% CI**	**P-value**
**Anti-cancer therapy**	-0.036	-0.066-(-0.0053)	0.021	-0.029	-0.055-(-0.0016)	**0.038**
**Age**	-0.11	-0.17-(-0.056)	**<0.001**	-0.11	-0.16-(-0.050)	**<0.001**
**Hematologic cancer**	0.28	-0.86-1.42	0.63	-0.33	-1.080-0.41	0.38

Supplementary Table 4. Univariate and multivariate meta-regression models of variables associated with serologic response after two doses of vaccine

	Univariate meta-regression			Multivariate meta-regression		
	**Coefficient**	**95% CI**	**P-value**	**Coefficient**	**95% CI**	**P-value**
**Anti-cancer therapy**	0.038	0.010-0.066	**0.0077**	0.012	-0.014-0.037	0.38
**Age**	-0.11	-0.33-0.11	0.32	-0.033	-0.17-(-0.47)	0.64
**Hematologic cancer**	-2.46	-3.45-(-1.48)	**<0.001**	-2.16	-3.26-(-3.86)	**<0.001**

Supplementary Table 5. Grading of Recommendations, Assessment, Development, and Evaluation (GRADE) criteria for studies included in the meta-analysis

(A) Meta-analysis of observational studies assessing serological response after two doses of vaccine

**Number of participants**	**Starting Level of Evidence**	**Quality assessment**					**Reasons to increase level of evidence (Large magnitude of effect; Dose-response gradient; Potential confounding)**	**Overall quality of evidence**
		**Risk of Bias**	**Inconsistency**	**Indirectness**	**Imprecision**	**Publication bias**		
1137	Low	Not serious	Not serious	Serious	Not serious	Not serious	N/A	Low

(B) Meta-analysis of case control studies comparing serological response after two doses of vaccine to controls

**Number of participants**	**Starting Level of Evidence**	**Quality assessment**					**Reasons to increase level of evidence (Large magnitude of effect; Dose-response gradient; Potential confounding)**	**Overall quality of evidence**
		**Risk of Bias**	**Inconsistency**	**Indirectness**	**Imprecision**	**Publication bias**		
903 (cases) and 320 (controls)	Low	Not serious	Serious	Serious	Not serious	Not serious	N/A	Low
